# Causal dynamic decision-making for robotic systems in non-Markovian high-difficulty surgery

**DOI:** 10.3389/fneur.2026.1767832

**Published:** 2026-02-20

**Authors:** Guo Na, Tan Minghui, Li Tiantian, Liu Yang, Zhang Qinjian, Li Yuanxin, Xu Tianlei, Sun Fuchun

**Affiliations:** 1 School of Computer and Communication Engineering, University of Science and Technology Beijing, Beijing, China; 2 College of Information and Electrical Engineering, China Agricultural University, Beijing, China; 3 School of Mechanical and Electrical Engineering, Beijing Information Science and Technology University, Beijing, China; 4 Beijing Tsinghua Changgung Hospital, Tsinghua University, Beijing, China; 5 Institute of Intelligent Healthcare, Tsinghua University, Beijing, China; 6 Department of Computer Science and Technology, Tsinghua University, Beijing, China

**Keywords:** causal inference, dynamic decision-making, Granger causality, non-Markov processes, surgical robotics

## Abstract

Markov assumption-based surgical decision models cannot account for the time-varying, irregular effects of high-risk intraoperative anomalies such as sudden hemorrhage or inadvertent instrument loss, making them inadequate for specialized procedures like neurosurgery and spinal interventions. To overcome the non-Markovian limitations of conventional surgical process modeling, this study develops a causal modeling framework based on Vector Autoregression (VAR) and Granger causality analysis. The framework constructs a causal chain (original gesture $S_{i}$ → abnormal event $E_{j}$ → recovery action $Z_{k}$ ) to enable intelligent response and adaptive decision-making. Validation was performed on a large-scale synthetic dataset containing 10,000 samples (including anomaly, positive, and negative cases), and evaluated using accuracy, F1-score, and recall metrics. Experimental results show the proposed method achieves 95.60% accuracy in causal inference, maintaining stability at 10,000 samples with an F1 score of 95.77%. Notably, recall (95.88%) slightly exceeds precision (95.34%), reflecting the clinical principle of prioritizing safety. The framework effectively captures non-Markovian temporal correlations induced by abnormal events, overcoming key limitations of traditional approaches. Its design is not procedure-specific, providing a versatile and generalizable pathway for enhancing autonomous decision-making in surgical robots across diverse clinical applications.

## 1 Introduction

Decision-making is the core prerequisite for surgical robots to achieve autonomous operation. Recent advances in embodied intelligence (1) have propelled the field beyond traditional leader-follower control toward systems capable of environmental perception and autonomous decision-making. The early STAR robot, developed by the Johns Hopkins University team, utilized near-infrared fluorescence imaging for millimeter-level tissue perfusion identification (2, 3); while the more recent SRT-H system employs miniature cameras and precision control to achieve submillimeter accuracy in vascular clamping (4, 5). And frameworks like VPPV have demonstrated “zero-shot transfer” of operational strategies for multi-task decision-making (1).

**Figure 1:**
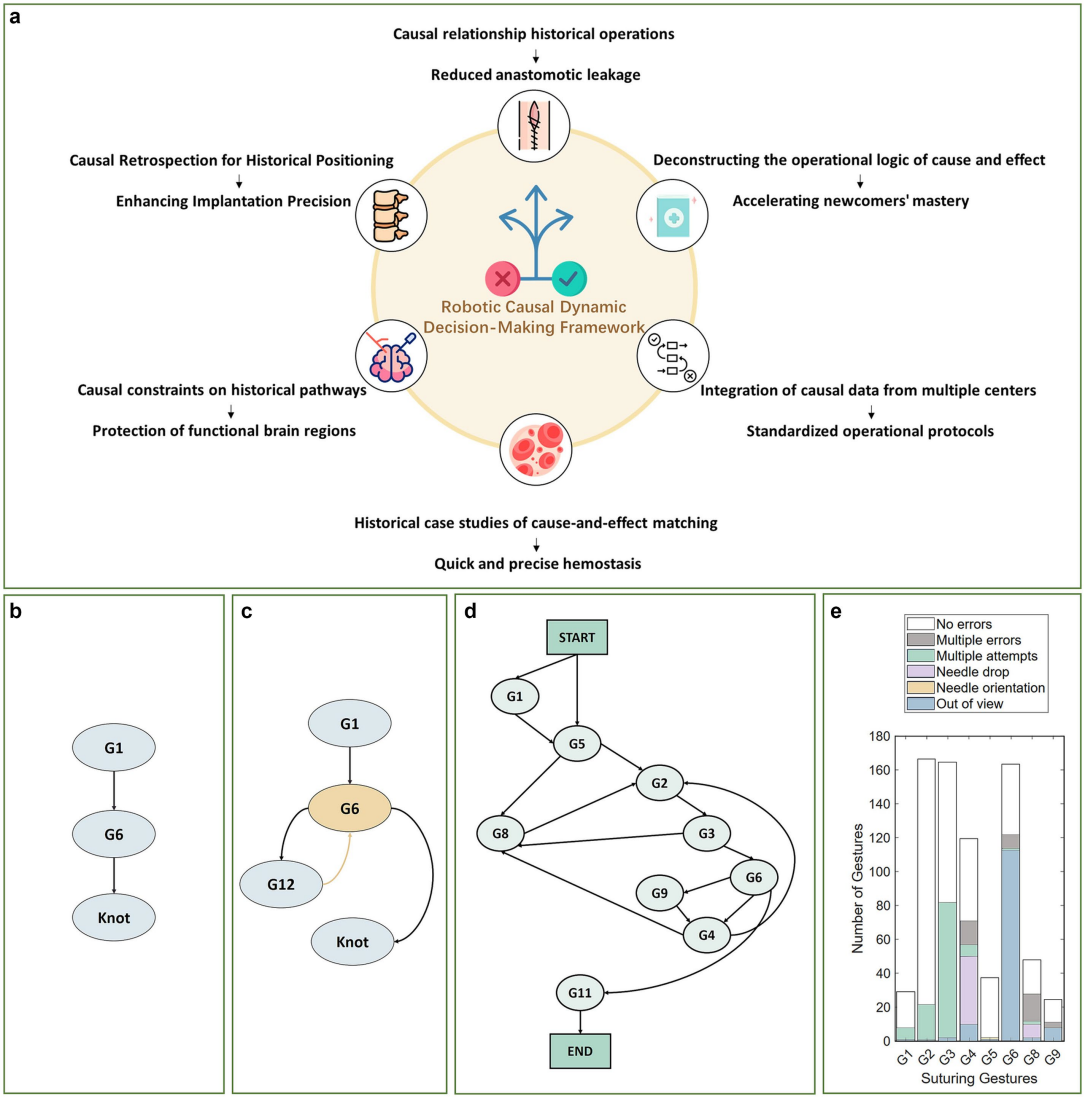
Clinical value and schematic representation of the causal dynamic decision-making framework for robotic surgery in non-Markovian high-difficulty scenarios. **(a)** Surgical application value of the causal dynamic decision-making framework; **(b)** Surgical suturing process under normal conditions; **(c)** Surgical suturing procedures in abnormal situations (such as needle drops); **(d)** Syntax diagram for surgical tasks (26); **(e)** Execution errors under abnormal circumstances (27).

However, existing surgical decision-making methods predominantly focus on conventional laparoscopic procedures such as cholecystectomy, with a primary emphasis on automating localized manipulations such as suturing and knot-tying. Systematic research into task-level autonomous decision-making for complex, high-risk surgical specialties including neurosurgery and spinal surgery remains notably scarce. Task-level surgical video data are often confounded by numerous variables such as instrument slippage (as visually illustrated for normal vs. abnormal scenarios Figures 1b,c) (6) and tissue deformation (7), which exhibit distinct non-Markovian temporal characteristics. This renders conventional temporal models inadequate for capturing long-range causal dependencies, thereby constraining the adaptability and reliability of current systems in complex surgical settings: Deterministic methods such as behavior trees (8, 9), behavior networks (10), or task priority graphs (11) are too rigid to adapt to unexpected events, while probabilistic models based on Markov Decision Processes (MDPs) (12, 13) fail to capture long-range causal relationships. Although the “Transformer-based planning + imitation/reinforcement learning-based control” paradigm (4, 14) is mainstream, traditional reinforcement learning often optimizes statistical correlations (15), leading to performance drops during Sim2Real transfer or sensor failure.

Causal reasoning, which transforms correlational strategies into causal ones (16, 17), provides new approaches to address partial observability (POMDP) and confounding factors in robotic perception. Work on Local Causal Models (LCMs) (18) and structural causal models (19, 20) has shown promise in improving sample efficiency and enabling zero-shot transfer in generic robotic tasks. However, these general causal inference methods are ill-suited for surgical scenarios: LCMs rely on locally factored dynamics and stationary environment assumptions, making them incapable of addressing abrupt, non-stationary abnormal events intraoperatively; structural causal models prioritize static causal graph construction, which hinders their ability to capture dynamic, long-term temporal dependencies arising from intraoperative disruptions. Consequently, they cannot be readily applied to surgical scenarios characterized by non-stationary state spaces and unforeseen anomalies. Surgical applications require models that enable safe, priority-driven dynamic causal inference under clinical logic constraints. Therefore, a solution specifically designed for the unique needs of surgical scenarios is urgently needed.

Inspired by work on local confounding detection (21) and adaptive methods (22) in non-stationary environments, this study models surgical robot decision-making as a sequential reasoning problem constrained by clinical logic. We propose a causal dynamic reasoning framework tailored to non-Markovian surgical environments, employing Granger causality inference (23, 24) as the core mechanism. By integrating it with the clinical logic of “surgical gesture – abnormal event – recovery action” and optimizing the lag order of the VAR model (25), the framework achieves safe, priority-based detection of dynamic abnormal events and supports autonomous decision-making.

This approach yields a framework with inherent generalization capabilities. By integrating the core Granger causality mechanism with transferable clinical logic (as schematically depicted in Figure 1a), this framework is not only directly applicable to diverse surgical scenarios such as neurosurgery and spinal procedures but also effectively addresses the non-Markovian dynamics and unexpected anomalies common in clinical practice.

## 2 Method

### 2.1 Framework overview and causal analysis

#### 2.1.1 Dynamic causal characteristic analysis of surgical adverse events

Surgical abnormal events (such as instrument drops or tissue damage) interrupt predefined operational workflows (see the surgical task syntax diagram in Figure 1d), and their dynamic adjustment strategies rely on accurate understanding of the “preceding actions - anomaly type - response operations” causal chain. Such anomalous events exhibit pronounced temporal long-range dependencies in non-Markovian environments and may be categorized into two primary types: (1) Procedural errors, manifested as omitted steps or sequential errors; (2) Execution errors, including positioning deviations and instrument loss of control, refer to failures in single-step operations. In minimally invasive surgical environments, such execution errors are particularly common due to constrained visual fields and anatomical variations, directly impacting operational effectiveness and procedural continuity.

Taking the suturing task as an example (as shown in Figure 1e), the handling logic for execution errors exhibits significant causal correlation characteristics. For simplification, it is assumed that each type of error occurs only once and can be successfully addressed:

- Needle tip positioning abnormality (G2): When positioning inaccuracy occurs, the system must repeat the positioning operation (G2) until successful before proceeding with tissue puncture (G3);
- Equipment Fall Incident (E1): Should instrument drop be detected during the left-hand suture pulling (G6) process, the retrieval operation shall take precedence, thereafter, return to G6 to continue the subsequent procedure;
- Equipment Out of Bounds Incident (E3): When the right hand is tightening the suture (G9), the instrument moves beyond the field of view, it is necessary to first adjust the instrument’s position, then decide whether to continue G9 or switch to other operations based on the actual situation.

Notably, these recovery pathways are dynamically determined by the interaction between anomaly type and the current operational context, emphasizing the requirement for autonomous systems to conduct real-time, context-aware causal reasoning in unstructured surgical environment.

#### 2.1.2 Dynamic inference framework for surgical procedures based on Granger causality testing

**Figure 2:**
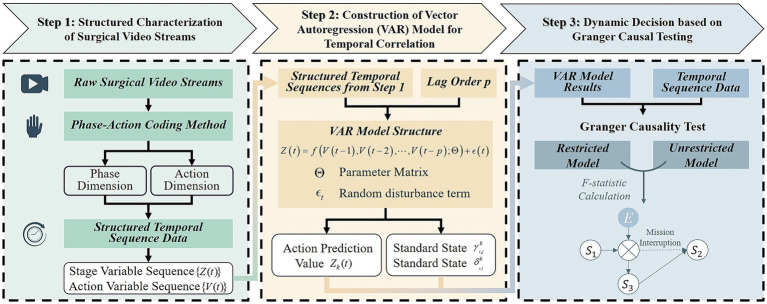
Implementation pipeline of the causal dynamic decision-making framework for surgical robots. Firstly, the key features of the surgical video are extracted to construct a characterization framework, and then the VAR model is used for temporal correlation modeling, and finally the causal timing is verified by Granger causality test, so as to accurately identify the causal relationship and provide theoretical support for the autonomous decision-making of surgical robots.

To establish a dynamic decision-making mechanism suitable for the non-Markovian surgical environments described above, this paper proposes a three-stage causal dynamic reasoning framework based on Granger causality testing. The overall implementation pathway of this framework is illustrated in Figure 2, which outlines the process from surgical video structuring to Granger causality verification. This framework is designed to identify causal relationships between surgical phases and actions, providing a basis for the dynamic decision-making of surgical robots. It comprises the following core components:

1. Structured representation of surgical video streams

Using deep learning models, key surgical phases and action markers are extracted from surgical videos to construct a structured representation framework. This approach addresses the challenge of modeling phase-action associations in complex scenarios, providing a robust data foundation for subsequent causal analysis.

2. Construction of vector autoregressive (VAR) models to capture temporal dynamics

A Vector Autoregression (VAR) model is applied to capture temporal correlations. Compared to traditional Markov models, the VAR model more effectively captures long-range dynamic dependencies inherent in non-Markovian surgical processes, enabling more comprehensive temporal analysis.

3. Dynamic decision based on granger causal testing

Granger causality testing is employed to validate genuine causal relationships by evaluating predictive capability differences. This method distinguishes between mere temporal correlations and true causal associations, providing a reliable autonomous decision-making strategy for surgical robots.

### 2.2 Structured characterization of surgical procedures

This study employs a “phase-action” coding method to convert continuous surgical videos into structured temporal sequences: the phase dimension is defined by the continuous execution of standard operational states together with sudden abnormal events; while the action dimension represents the executable operations of the robotic system at the current moment. This integrated approach aligns with surgical cognitive patterns, clearly distinguishing routine operations from abnormal events.

Let there be N standard operational states $S_{i}$ , M random events $E_{j}$ , W executable operations $Z_{k}$ , which are defined as follows:

At any time *t*:

$S_{i}(t)(i=1,2,\cdots ,N)\in \{0,1\}$ denotes whether the *i*-th standard operational state is active. If $S_{1}$ =‘*Needle Holding*’, $S_{1}(t)=1$ indicates that the step ‘*Needle Holding*’ is being performed, and $S_{1}(t)=0$ indicates it is not.

$E_{j}(t)(j=1,2,\cdots ,M)\in \{0,1\}$ denotes whether the *j*-th random event occurs. If $E_{1}$ = ‘*Needle Dropping*’, $E_{1}(t)=1$ indicates that the abnormal event ‘*Needle Dropping*’ has occurred, and $E_{1}(t)=0$ indicates it has not.

$Z_{k}(t)(k=1,2,\cdots ,W)\in \{0,1\}$ denotes whether the *k*-th executable action is triggered or recommended by the system. If $Z_{1}$ = ‘*Needle Retrieval*’, $Z_{1}(t)=1$ indicates that the action is triggered, and $Z_{1}(t)=0$ indicates it is not.

Accordingly, the surgical phase and action at time *t* can be described as:

$$V(t)={\left[S_{1}(t),S_{2}(t),\cdots ,S_{N}(t);E_{1}(t),E_{2}(t),\cdots ,E_{M}(t)\right]}^{T}$$

$$Z(t)={\left[Z_{1}(t),Z_{2}(t),\cdots ,Z_{W}(t)\right]}^{T}$$

Where, V(t) represents the stage variable at time t , integrating both standard operational states and random events. Z(t) denotes the action variable at time t , comprising W executable operational states $Z_{1}(t),Z_{2}(t),\cdots ,Z_{W}(t)$ .

### 2.3 Construction of vector autoregression (VAR) models

The core objective of this study is to learn a dynamic decision function from surgical video time-series data that can accurately describe the mapping from historical phase information to the current execution action. This function represents the conditional probability distribution of the system taking a specific action given the historical surgical context, which can be generally expressed as:

P(Z(t)∣V(t−1),V(t−2),⋯,V(t−p))

Where p denotes the length of the historical time window influencing the current decision. This conditional probability distribution essentially defines a state transition process, mapping the sequence of past p phase states {V(t−1),V(t−2),⋯,V(t−p)} to the action variable Z(t) at time t.

To characterize dynamic interactions within the ‘phase’ variable V(t) and its potential influence on the ‘action’ variable (t) , this study employs a Vector Autoregressive (VAR) model as the core temporal modeling tool. The VAR model enables simultaneous estimation of the interdependencies among multiple endogenous variables and captures the persistent effects of historical states on the current system through the introduction of lag terms. This provides an ideal structural foundation for subsequently identifying the Granger causal relationships between the “phase” and “action” variables.

The VAR-based phase-action model can be expressed as follows:

Z(t)=f(V(t−1),V(t−2),⋯,V(t−p);Θ)+ε(t)

Where, f(⋅) is a linear function, Θ is the parameter matrix to be estimated, and ε(t) is a random disturbance term. This model represents action decisions as a linear combination of historical phase variables, providing interpretable parameter estimates for subsequent analysis.

More specifically, each action variable (in Section 2.2) can be written as:

$$Z_{k}(t)=\sum_{q=1}^{p}\sum_{i=1}^{N}\gamma_{k,i}^{q}S_{i}(t-q)+\sum_{q=1}^{p}\sum_{j=1}^{M}\delta_{k,j}^{q}E_{j}(t-q)+\varepsilon_{k}(t)$$

Where, $Z_{k}(t)\;(k=1,2,\cdots ,W)$ denotes the k-th action at time t , p is the lag order, representing the length of historical information influencing the current action, $\gamma_{k,i}^{q}$ represents the influence of the i-th state at lag q on action $Z_{k}(t)$ , $S_{i}(t-q)$ (i=1,2,⋯,N) is the value of the i-th phase variable at past time t−q , $\delta_{k,j}^{q}$ represents the influence of the j-th random event at lag q on action $Z_{k}(t)$ , $E_{j}(t-q)$ (j=1,2,⋯,M) is the value of the j-th random event at past time t−q , $\varepsilon_{k}(t)$ is a random noise term at time t .

This formulation captures both the influence of historical phase states and abnormal events on current actions, providing a clear, interpretable framework for modeling surgical decision-making. These actions may be triggered by abnormal events (e.g., “Bleeding” triggering “Electrocoagulation”) or arise naturally from routine surgical logic (e.g., the “Suturing” state leading to “Knot Tying”). Granger causality, which will be introduced in Section 2.4, is later incorporated into the time-series modeling framework to identify and remove spurious correlations, enabling the construction of a decision model with explicit causal interpretability and strong generalization.

### 2.4 Dynamic decision based on Granger causal testing

Building upon the VAR-based phase-action model introduced in Section 2.3, Granger causality tests are employed to identify temporal causal relationships between surgical phases and robotic actions. Herein, the “phase component” refers to $V_{\mathrm{ij}}(t)=(S_{i}(t),E_{j}(t))$ , which denotes the standard operational state variable $S_{i}(t)$ or individual random event variable $E_{j}(t)$ extracted from the integrated stage variable V(t) (defined in Section 2.2). In a non-Markovian surgical environment, if incorporating historical information from past phase variables $S_{i}(t-1),S_{i}(t-2),\cdots ,S_{i}(t-q)$ and random events $E_{j}(t-1),E_{j}(t-2),\cdots ,E_{j}(t-q)$ significantly improves the prediction of the current action $Z_{k}(t)$ , these variables are considered Granger causes of $Z_{k}(t)$ . The testing process, detailed in Algorithm 1, is performed independently for each potential (phase component, action component) causal pair.

Algorithm 1 Element-wise Granger causality testing in temporal sequences.
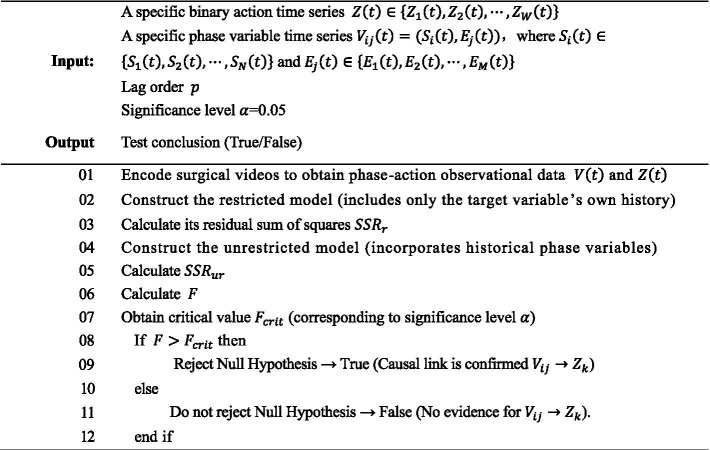

(1) Restricted model: Uses only the historical data of action $Z_{k}(t)$ itself for prediction, disregarding the influence of historical phase information, which can be expressed as follows:
$$Z_{k}(t)={\bar{\alpha }}_{0}+\sum_{q=1}^{p}{\bar{\beta }}_{k}^{q}Z_{k}(t-q)+\varepsilon_{k}(t)$$

Here, ${\bar{\alpha }}_{0}$ denotes the constant term, p represents the lag order, ${\bar{\beta }}_{k}^{q}$ signifies the autoregressive coefficient of $Z_{k}(t)$ at lag q.

(2) Unrestricted model: Incorporates lagged terms of all historical phase variables to explicitly capture the respective causal influences of past states and events on the current action.
$$\begin{array}{c} Z_{k}(t)=\alpha_{0}+\sum_{q=1}^{p}\beta_{k}^{q}Z_{k}(t-q)+\sum_{q=1}^{p}\sum_{i=1}^{N}\gamma_{k,i}^{q}S_{i}(t-q)\\ +\sum_{q=1}^{p}\sum_{j=1}^{M}\delta_{k,j}^{q}E_{j}(t-q)+\varepsilon_{k}(t) \end{array}$$

The coefficient $\gamma_{k,i}^{q}$ and $\delta_{k,j}^{q}$ represent the average effect of the state $S_{i}(t-q)$ and $E_{j}(t-q)$ has on the action $Z_{k}(t)$ at lag q, e.g., the driving effect of a G6 operation on the picking up needle action, or the triggering effect of dropping needle on the picking up needle action.

(3) Causal significance F

The likelihood ratio statistic is used to assess whether the predictive improvement of the unrestricted model relative to the restricted model is statistically significant, which can be expressed as follows:

$$F=\frac{(\mathrm{SS}R_{r}-\mathrm{SS}R_{\mathrm{ur}})/Q}{\mathrm{SS}R_{\mathrm{ur}}/(n-p-1)}$$

Here, $\mathrm{SS}R_{r}$ and $\mathrm{SS}R_{\mathrm{ur}}$ represent the sums of squared residuals of the restricted and unrestricted models, respectively; $\mathrm{SS}R_{r}$ reflects the prediction error without causal variables, while $\mathrm{SS}R_{\mathrm{ur}}$ reflects the prediction error after introducing causal variables. n denotes the sample size, Q represents the total number of lagged terms of the causal variables, p is the lag order (Q = 2p).

Based on the results of the Granger causality test, a dynamic correction mechanism for surgical procedures is established. When the system detects an abnormal event, it automatically triggers the corresponding corrective action. For example, “pick up needle + re-execute G6” can be determined by integrating its causal association “dropped needle” with the preceding state “G6 operation.” Unlike conventional predefined workflows, this mechanism generates adaptive response strategies through data-driven causal inference, enabling process reconstruction in non-sequential or unexpected surgical scenarios. The corresponding pseudocode is presented as follows:

Algorithm 2 Dynamic correction mechanism. 
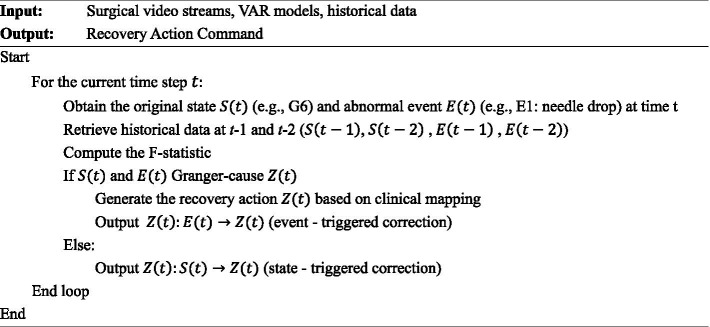

## 3 Results

This section employs synthetic data to simulate clinically abnormal scenarios, thereby quantitatively evaluating the causal reasoning framework’s capability to dynamically model the sequential logic of surgical procedures.

### 3.1 Dataset

Due to the scarcity of annotated data on abnormal events during actual surgical procedures, coupled with ethical and privacy constraints, this study employs synthetic data to validate a surgical process modeling approach based on Granger causality testing. The dataset design focuses on the causal relationship between “abnormal events” and “countermeasures,” comprising three core temporal variables:

$S_{i}$ (original gesture, e.g., G6 “left hand pulling thread”).

$E_{j}$ (abnormal event, e.g., “dropped needle”).

$Z_{k}$ (recovery action, e.g., “pick up needle + re-execute G6”).

A variable value of 1 indicates that an event has occurred, while 0 indicates that it has not.

This study employs both positive and negative examples as samples. Positive examples are constructed based on clinical logic, establishing explicit causal relationships such as “needle drop → needle retrieval” and “inaccurate positioning → repositioning.” This ensures that $Z_{k}$ is driven by historical data from $S_{i}$ and $E_{j}$ , thereby simulating the anomaly handling procedures encountered in actual surgical procedures. Negative examples are generated by randomly producing sequences of unrelated variables ( $S_{i}$ , $E_{j}$ , $Z_{k}$ )to eliminate temporal correlation interference, thereby validating the model’s discriminative capability in scenarios lacking genuine causal relationships.

To enhance the clinical plausibility of synthetic data, three senior consultants reviewed all predefined causal relationships, yielding a Kappa consistency score of 0.92. Furthermore, to better simulate real surgical environments, we introduced clinical noise in 20% of samples (specifically simulating gesture recognition bias caused by tissue occlusion) to bolster both data authenticity and model robustness. In this study, two datasets of different scales were constructed: one containing 5,000 samples (2,500 positive and 2,500 negative), and the other containing 10,000 samples (5,000 positive and 5,000 negative). The goal is to evaluate the model’s stability and generalization ability under varying data scales.

### 3.2 Experimental setup

When constructing the VAR model, the selection of the lag order *p* is crucial to model performance. To effectively capture the influence of prior actions on the current recovery operation while avoiding excessive noise, *p* must be properly determined. Considering that intraoperative abnormal events typically span about two action steps from occurrence to response (for example, after a “needle drop,” the surgeon must first “pick up the needle” and then “rethread”), this study preliminarily sets the lag order to *p* = 2.

To further optimize the selection of the lag order and improve both reproducibility and transparency, the Akaike Information Criterion (AIC) was adopted for validation. As a widely recognized information-theoretic metric for model selection, the AIC effectively balances goodness of fit with model parsimony. Specifically, lower AIC values indicate a more optimal trade-off between capturing meaningful temporal dependencies and minimizing the risk of overfitting, thereby ensuring the model remains both robust and computationally efficient. By comparing AIC values under different *p* settings, the results show that when *p* = 2, the model achieves the smallest residual (AIC = −3.2), outperforming *p* = 1 (AIC = −2.8) and *p* = 3 (AIC = −2.9). Therefore, the optimal lag order was finally determined to be *p* = 2, ensuring an accurate representation of the surgical dynamics while avoiding overfitting.

The experimental parameters are set as follows: the sample size *n* = 5,000, the lag order *p* = 2, the generation probabilities for the variables $S_{i}$ (normal gesture) and $E_{j}$ (abnormal event) are 0.3 and 0.2, respectively, and the weighting coefficient for the lagged term S and that for the lagged term E are both 0.5. The regression coefficients (including $\alpha_{0}$ , $\beta_{i}$ , $\gamma_{j}$ , and $\delta_{l}$ ) are estimated using Ordinary Least Squares (OLS), with a total of Q = 4 lag terms.

### 3.3 Experimental results

**Figure 3:**
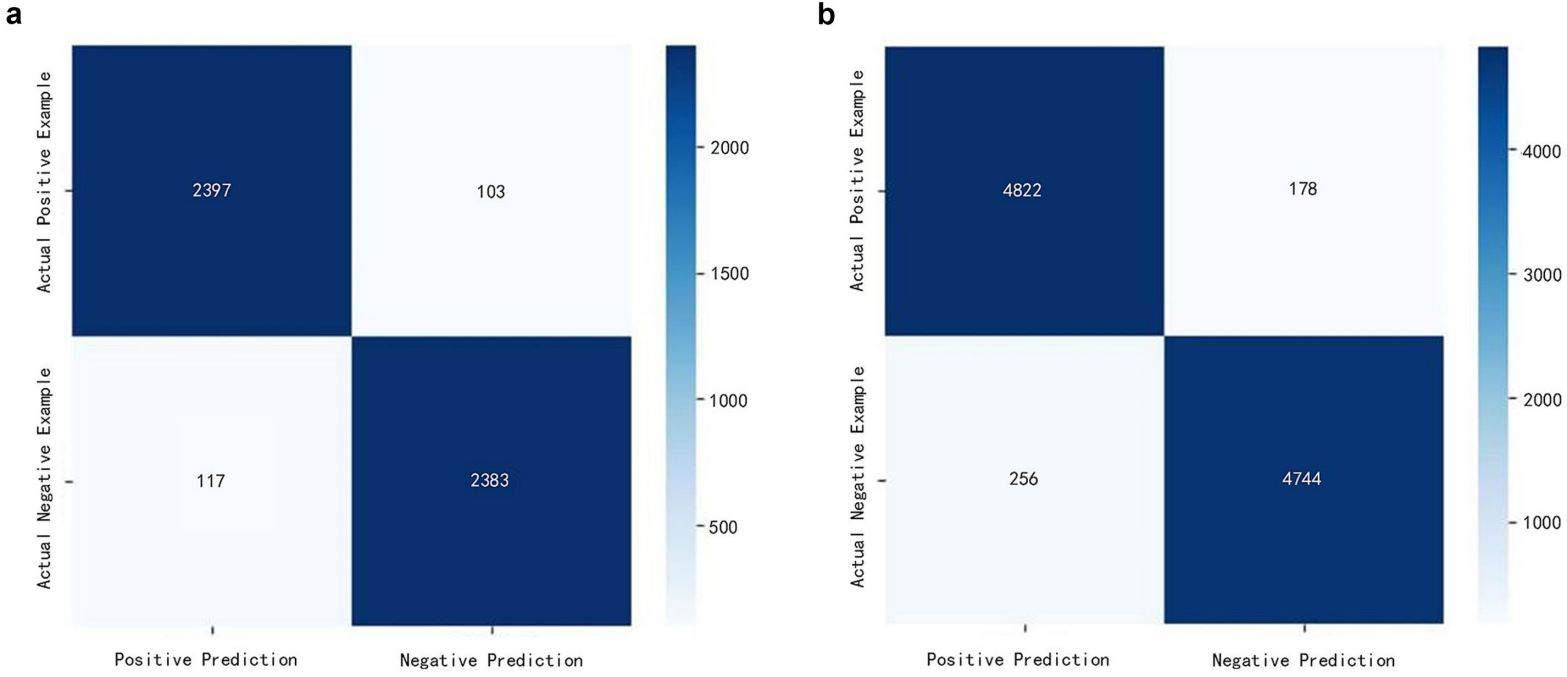
Confusion matrix analysis for different sample sizes. **(a)** The results of the model at a scale of 5,000 samples (2,500 positives and 2,500 negatives); **(b)** the results of the model at a scale of 10,000 samples (5,000 positives and 5,000 negatives).

Table 1 and Figure 3 demonstrate the performance of the proposed method across varying sample sizes. The model achieved an accuracy exceeding 95.6% on both 5,000 and 10,000 samples, with the Matthews correlation coefficient stabilizing at 0.912. This indicates excellent discriminative capability and generalization performance.

**Table 1:** Analysis of experimental results.

Positive example\Negative example	Accuracy rate	Precision rate	Recall rate	F1 score	MCC
2,500\2,500	95.60%	95.34%	95.88%	95.60%	0.912
5,000\5,000	95.66%	94.96%	96.44%	95.77%	0.912

It is worth noting that the model’s recall consistently exceeds its precision, indicating a preference for minimizing the rate of missed true causative events—that is, reducing false negatives. In safety-critical surgical scenarios, this bias toward “better to err on the side of false positives than false negatives” aligns with the safety-first clinical principle. It helps ensure all critical anomalies are effectively identified and trigger appropriate response mechanisms.

## 4 Discussion

In high-complexity surgical scenarios such as neurosurgery and spinal surgery, confounding factors including instrument slippage, tissue deformation (6, 7), and visual field occlusion embedded in task-level video data exhibit distinct characteristics of cross-phase correlation and dynamic coupling over the temporal dimension. The non-Markovian nature shaped by these long-range causal dependencies profoundly underscores the inherent complexity of unstructured surgical environments, emerging as a key bottleneck that hinders surgical robots from achieving high-level autonomous decision-making. However, traditional temporal modeling methods are constrained by short-range dependency assumptions (8, 12), impeding their ability to effectively capture such long-range causal structures; meanwhile, existing systems based on deterministic rules or Markov Decision Processes (MDPs) fail to dynamically respond to and adaptively adjust for process disruptions induced by abnormal events, primarily due to their rigid architectures and inadequate state modeling. Ultimately, this severely compromises the reliability, robustness, and clinical applicability of these systems in real-world complex surgical settings.

Inspired by local causality discovery and non-stationary adaptive learning theories (21, 22), this study proposes a dynamic reasoning framework based on Granger causality testing. This framework translates the clinical logic of “surgical gesture–abnormal event–recovery action” into computable temporal causal hypotheses. Through vector autoregressive modeling and Granger significance testing, it achieves data-driven identification of abnormal causal chains and dynamic decision-making.

Experimental results demonstrate that the framework achieves excellent and stable performance on synthetic datasets. The model demonstrated accuracy exceeding 95.6% and a MCC value of 0.912 across both 5,000 and 10,000 sample sizes. Notably, when the sample size increased to 10,000 instances, the F1 score remained at 95.77%, attesting to its exceptional stability and robust capability to distinguish genuine causality from coincidental correlations. The core advantage of this approach lies in constructing causal chains linking “original gesture-abnormal event-recovery action” (e.g., “dropping needle → picking up needle → resuming threading”). This not only captures the direct association between “abnormal event → countermeasure” but also quantifies the dynamic influence of historical states on current decisions through the VAR model’s lagged term coefficients. Consequently, it overcomes the traditional Markov model’s reliance on “fixed temporal sequences”. Crucially, the model exhibits a persistent tendency for recall (95.88%) to exceed precision (95.34%), reflecting a cautious bias toward prioritizing “better to report a false positive than miss a true positive.” In safety-critical surgical scenarios, this design philosophy—which minimizes false negatives (i.e., missed detection of abnormal causal pairs)—aligns closely with the safety-first clinical principle. It effectively ensures that all critical anomalies (such as “dropped needles”) are identified and trigger appropriate responses, thereby significantly enhancing the system’s inherent safety. The confusion matrix further confirms that the model’s false negative rate consistently remains below 5%, demonstrating substantial application potential in surgical anomaly modeling and autonomous response.

Despite these initial advances, several limitations persist that warrant further investigation. First, model validation currently relies on synthetic data; while high performance is achieved under simplified assumptions, the framework may encounter generalization challenges in real surgical environments. Inherent complexities including continuous tissue deformation, variable instrument–tissue interactions such as context-dependent friction or adhesion, and instrument occlusion introduce unmodeled dynamic noise that is not fully captured in synthetic datasets, potentially undermining the framework’s reliability during clinical transition. To bridge the gap between simulation and actual clinical practice, we plan to collaborate with clinical institutions to collect and annotate real surgical videos across specialties such as neurosurgery and spinal surgery that include intraoperative anomalies. Establishing a high-quality dataset requires satisfying two core criteria, namely that the video library must cover an extensive spectrum of surgical scenarios to ensure robust representativeness, and that comprehensive documentation of surgical phases, potential anomalous events, and their respective mitigation strategies must be integrated. To meet these requirements, experienced surgeons will conduct systematic reviews of surgical footage to generate descriptive narratives, which will then be parsed and structured by large language models to refine classification systems for standard operative states and abnormal events. Following the formulation of rigorous annotation protocols, the labeling will be independently completed by multiple professionals, with inter-annotator consistency verified via the Kappa coefficient to guarantee the dataset’s reliability and generalizability. Furthermore, the current Vector Autoregressive (VAR) model may struggle to capture the extended temporal dependencies inherent in complex procedures. Consequently, future work will explore incorporating advanced architectures such as Transformers to enhance the modeling of long-range causal relationships and further improve decision-making robustness.

In summary, this study addresses the critical issue of long-range dependencies triggered by abnormal events in non-Markovian surgical environments through a novel causal dynamic reasoning framework. By integrating Granger causality testing with vector autoregressive models, the proposed method successfully constructs interpretable “surgical gesture-abnormal event-recovery action” chains, enabling autonomous reasoning and dynamic decision-making. The framework’s high accuracy, stability, and inherent bias toward safety—evidenced by recall consistently exceeding precision—provide a robust and clinically-aligned foundation for handling surgical disruptions. The insights and architecture presented here mark a significant step forward in propelling surgical robots from programmed execution toward cognitive, adaptive decision-making.

## Data Availability

The raw data supporting the conclusions of this article will be made available by the authors, without undue reservation.

## References

[1] Long Y Lin A Kwok DHC Zhang L Yang Z Shi K . Surgical embodied intelligence for generalized task autonomy in laparoscopic robot-assisted surgery. Sci Robot. (2025) 10:eadt3093. doi: 10.1126/scirobotics.adt3093, 40668896

[2] Shademan A Decker RS Opfermann JD Leonard S Krieger A Kim PCW. Supervised autonomous robotic soft tissue surgery. Sci Transl Med. (2016) 8:337ra64. doi: 10.1126/scitranslmed.aad9398, 27147588

[3] Shakir T Atraszkiewicz D Hassouna M Pampiglione T Chand M. Beyond diagnosis: how advanced imaging technologies are shaping modern surgery. Artif Intell Surg. (2025) 5:270–82. doi: 10.20517/ais.2024.79

[4] Kim JWB Chen JT Hansen P Shi LX Goldenberg A Schmidgall S . SRT-H: a hierarchical framework for autonomous surgery via language-conditioned imitation learning. Sci Robot. (2025) 10:eadt5254. doi: 10.1126/scirobotics.adt5254, 40632876

[5] Haworth J Biswas R Opfermann J Kam M Wang Y Pantalone D (2024). Autonomous robotic system with optical coherence tomography guidance for vascular anastomosis. arXiv. Available online at: https://arxiv.org/abs/2410.07493 (Accessed July 5, 2025)

[6] Tapper A Leale D Megahan G Nacker K Killinger K Hafron J. Robotic instrument failure—a critical analysis of cause and quality improvement strategies. Urology. (2019) 131:125–9. doi: 10.1016/j.urology.2019.02.052, 31158353

[7] Monji-Azad S Kinz M Kothari S Khanna R Mihan AC Männel D . DefTransNet: a transformer-based method for non-rigid point cloud registration in the simulation of soft tissue deformation. Meas Sci Technol. (2025) 36:076006. doi: 10.1088/1361-6501/ade613

[8] Colledanchise M Parasuraman R Ögren P. Learning of behavior trees for autonomous agents. IEEE Trans Games. (2019) 11:183–9. doi: 10.1109/TG.2018.2816806

[9] Hu D Gong Y Hannaford B Seibel EJ. Semi-autonomous simulated brain tumor ablation with RAVENII surgical robot using behavior tree. Proc IEEE Int Conf Robot Autom. (2015):3868–75. doi: 10.1109/ICRA.2015.7139738 PMC4578323 26405563

[10] Hayes B Scassellati B. Autonomously constructing hierarchical task networks for planning and human-robot collaboration. Proc IEEE Int Conf Robot Autom. (2016):5469–76. doi: 10.1109/ICRA.2016.7487760

[11] Pardowitz M Knoop S Dillmann R Zollner RD. Incremental learning of tasks from user demonstrations, past experiences, and vocal comments. IEEE Trans Cybern. (2007) 37:322–32. doi: 10.1109/tsmcb.2006.886951, 17416160

[12] Zhang J Dridi M El Moudni A. Scheduling elective surgeries with Markov decision process and approximate dynamic programming. IFAC-PapersOnLine. (2019) 52:1831–6. doi: 10.1016/j.ifacol.2019.11.468

[13] Alison B Brown L. BJS.03 Markov decision analysis of treatment pathways for resectable malignancy of the oesophagus or gastrooesophageal junction. Br J Surg. (2021) 108:znab310.005. doi: 10.1093/bjs/znab310.005

[14] Kim JW Zhao TZ Schmidgall S Deguet A Kobilarov M Finn C (2024). Surgical robot transformer (SRT): imitation learning for surgical tasks. arXiv Available online at: https://arxiv.org/abs/2407.12998 (Accessed July 8, 2025)

[15] Zeng Y Cai R Sun F Huang L Hao Z. A survey on causal reinforcement learning. IEEE Trans Neural Netw Learn Syst. (2025) 36:5942–62. doi: 10.1109/TNNLS.2024.3403001, 40030342

[16] Bareinboim E Pearl J. Causal inference and the data-fusion problem. Proc Natl Acad Sci USA. (2016) 113:7345–52. doi: 10.1073/pnas.1510507113, 27382148 PMC4941504

[17] Dasgupta I Wang J Chiappa S Mitrovic J Ortega P Raposo D (2019). Causal reasoning from meta-reinforcement learning. arXiv. Available online at: https://arxiv.org/abs/1901.08162 (Accessed July 8, 2025)

[18] Pitis S Creager E Garg A. "Counterfactual data augmentation using locally factored dynamics" In: Larochelle H Ranzato M Hadsell R Balcan MF Lin H, editors. Advances in neural information processing systems 33 (2020). 3976–90.

[19] Buesing L Weber T Zwols Y Racaniere S Guez A Lespiau JB (2018). Woulda, coulda, shoulda: counterfactually-guided policy search. arXiv. Available online at: https://arxiv.org/abs/1811.06272 (Accessed October 10, 2025).

[20] Mozifian M Zhang A Pineau J Meger D. (2020). Intervention design for effective Sim2Real transfer. arXiv. Available online at: https://arxiv.org/abs/2012.02055 (Accessed July 10, 2025)

[21] Seitzer M Schölkopf B Martius G. "Causal influence detection for improving efficiency in reinforcement learning" In: Ranzato M Beygelzimer A Dauphin Y Liang PS Vaughan JW, editors. Advances in neural information processing systems 34 (2021). 22905–18.

[22] Feng F Huang B Zhang K Magliacane S. "Factored adaptation for non-stationary reinforcement learning" In: Koyejo S Mohamed S Agarwal A Belgrave D Cho K Oh A, editors. Advances in neural information processing systems. Red Hook, NY, USA: Curran Associates, Inc. (2022) 35, 31957–71.

[23] Granger CWJ. Testing for causality: a personal viewpoint. J Econ Dyn Control. (1980) 2:329–52. doi: 10.1016/0165-1889(80)90069-X

[24] Granger CWJ. Investigating causal relations by econometric models and cross-spectral methods. Econometrica. (1969) 37:424–38. doi: 10.2307/1912791

[25] Okada T Okamoto S Yamada Y. Affective dynamics: causality modeling of temporally evolving perceptual and affective responses. IEEE Trans Affect Comput. (2022) 13:628–39. doi: 10.1109/TAFFC.2019.2942931

[26] Shao R Lan X. Adversarial auto-encoder for unsupervised deep domain adaptation. IET Image Process. (2019) 13:2772–7. doi: 10.1049/iet-ipr.2018.6687

[27] Li H Liu H Fu H Xu Y Shu H Niu K . A generic fundus image enhancement network boosted by frequency self-supervised representation learning. Med Image Anal. (2023) 90:102945. doi: 10.1016/j.media.2023.102945, 37703674

